# Translation and cultural adaptation of IPOS (integrated palliative care outcome scale) in Estonia

**DOI:** 10.1186/s41687-021-00288-z

**Published:** 2021-01-28

**Authors:** Merli Laissaar, Riina Hallik, Pille Sillaste, Ulvi Ragun, Mari-Leen Pärn, Kaiu Suija

**Affiliations:** 1 Tallinn University of Technology, Ehitajate tee 5, 19086 Tallinn, Estonia; 2 North Estonia Medical Centre Foundation, J. Sütiste tee 19, 13419 Tallinn, Estonia; 3 Tartu University Hospital, L. Puusepa 1a, 50406 Tartu, Estonia

**Keywords:** Patient-reported outcome measure, IPOS, Estonia, Palliative care, Patient reported outcome

## Abstract

**Background:**

Patient Reported Outcome Measures (PROMs) are questionnaires that could be used in palliative care (PC) to evaluate patient well-being and monitor their care. PROMs enable a focus on what is important to patients by putting the patient at the center of care. Adapting an existing PROM requires considering cultural differences, general usability and understandability of translated terms.

**Aim:**

To translate and culturally adapt both the patient and staff three and seven day versions of the Integrated Palliative care Outcome Scale (IPOS) into Estonian. The IPOS consist of 10 questions (staff versions 9 questions) and 17 close ended items. The sub aim is to describe the differences and discrepancies found during the adaptation process and compare the results with previous research.

**Methods:**

The translation and adaptation process of IPOS was conducted using recommended guidelines consisting of six phases and included cognitive interviews with patients (*n* = 11) and palliative care multidisciplinary team members (*n* = 8). The study was conducted in two major Estonian hospitals.

**Results:**

The Estonian IPOS demonstrated face and content validity, acceptance by patients and staff. As a result of expert group review and cognitive interviews with patients and staff, 9 semantic changes were implemented.

**Conclusions:**

Patient and staff versions of the IPOS with a recall of 3 or 7 days were translated and culturally adapted for Estonia. The Estonian IPOS four versions are ready for use in Estonia.

## Background

Palliative care (PC) is an approach with the main goal to improve the health related quality of life (HRQL) of patients and their families in situations with a life-threatening illness by ensuring physical comfort with timely treatment of pain, and relief from other sensitive areas such as psychosocial and spiritual satisfaction and well-being [1]. In the absence of curative treatment, the objective is to promote daily coping of patients by improving and maintaining the HRQL [1]**.**

Patient centred outcome measurement plays a relevant role in assessing needs, care and well-being in PC settings [2–5]. Patient Reported Outcome Measures (PROM) are tools to measure patient outcomes from the patient perspective [3]. Assessing HRQL patient-centred outcomes (i.e. asking patients to assess their physical, social, psychological and spiritual condition) is one of the only ways to research and ensure effective and best possible PC [2]. Measures used in PC should be multidimensional and provide an overview of the physical symptomatology (pain, drowsiness, etc.) and the psychological, social and spiritual condition of the patient [2, 3].

There is currently no nationally accepted suitable holistic patient-centred measure or method available in Estonia for the assessment of PC patient needs. Developing a new measure in Estonia would be extremely resource and cost-intensive and unreasonable, given the small population of the country. Among the numerous reliable and validated measures currently used in the world, it would be rational to implement internationally recognized and validated measure developed specifically for PC (to measure symptoms and other PC concerns perceived by the patients). In addition to assessing patient-centred outcomes, this would also allow future participation in international studies and analysis of data collected on similar grounds. Important aspects to consider in the choice of PROM are cultural adaption, comparison possibilities in national as well as international level, usage availability for both patient and PC team members and information-intensive yet brief in style [2]. Adoption of an existing measure should consider the cultural differences between countries; general usability and understandability of translated terms and be done in a scientific standard [6].

Palliative care Outcome Scale (POS) is a PROM, which was initially developed by Professor Irene Higginson of King’s College London in 1999 [7]. Since then, the tool has been translated to many languages and validated in various settings including hospitals, nursing homes and hospices [2, 8–11]. Several additional (combined) questionnaires have been developed based on the initial POS measure (also still used today) [4, 9, 12] such as the POS Symptom list (POS-S), which is a questionnaire for symptom information capturing [9].

The IPOS questionnaire is available for patients and staff (when patient is unable to self-report) and for two time periods: past three days and past week [9]. IPOS is a combined measure based on the POS, POS-S and APCA Africa POS measures. The APCA Africa POS is a variation that has been designed and validated for Africa. The IPOS consists of questions assessing the symptoms, anxiety and affective disorders of the patient as well as their general well-being and satisfaction, need for information and practical concerns [4, 9]. The measure is multidimensional, allowing the assessment of the condition of the person as a whole while also considering their assessment on their physical, social, psychological and spiritual condition [4]. The questionnaire is simple, understandable and usable in all healthcare institutions providing PC. The staff does not require special training to use the questionnaire, and it can be filled in quickly by both patients and staff members [3, 9]. The IPOS questionnaire has been widely used in studies and in clinical practice [3, 9, 13–16]. The first question is open-ended and asked for the main problems or concerns of the patient over the past three or seven days (depending whether the questionnaire with recall time three or seven days is used). The IPOS includes17 closed-ended items administered using five category response optionss. The first 10 close-ended items assess symptoms. Following these items, the respondent is asked to list up to three other symptoms. This is followed by three Items (Q3-Q5) assessing anxiety and depression; three items assessing general well-being and satisfactions, and an item asking about practical problems. The last question asks: “How did you complete this questionnaire?” In the staff version of the questionnaire, item two regards to symptoms includes an option “Cannot assess” and item 10 “How did you complete this questionnaire?” has been omitted [9].

### Aim

Aim of the study is to translate and culturally adapt both the patient and staff versions of IPOS for Estonia. The subaim is to describe the differences and discrepancies found during the adaptation process and compare the results with the previous research.

## Methods

**Fig. 1:**
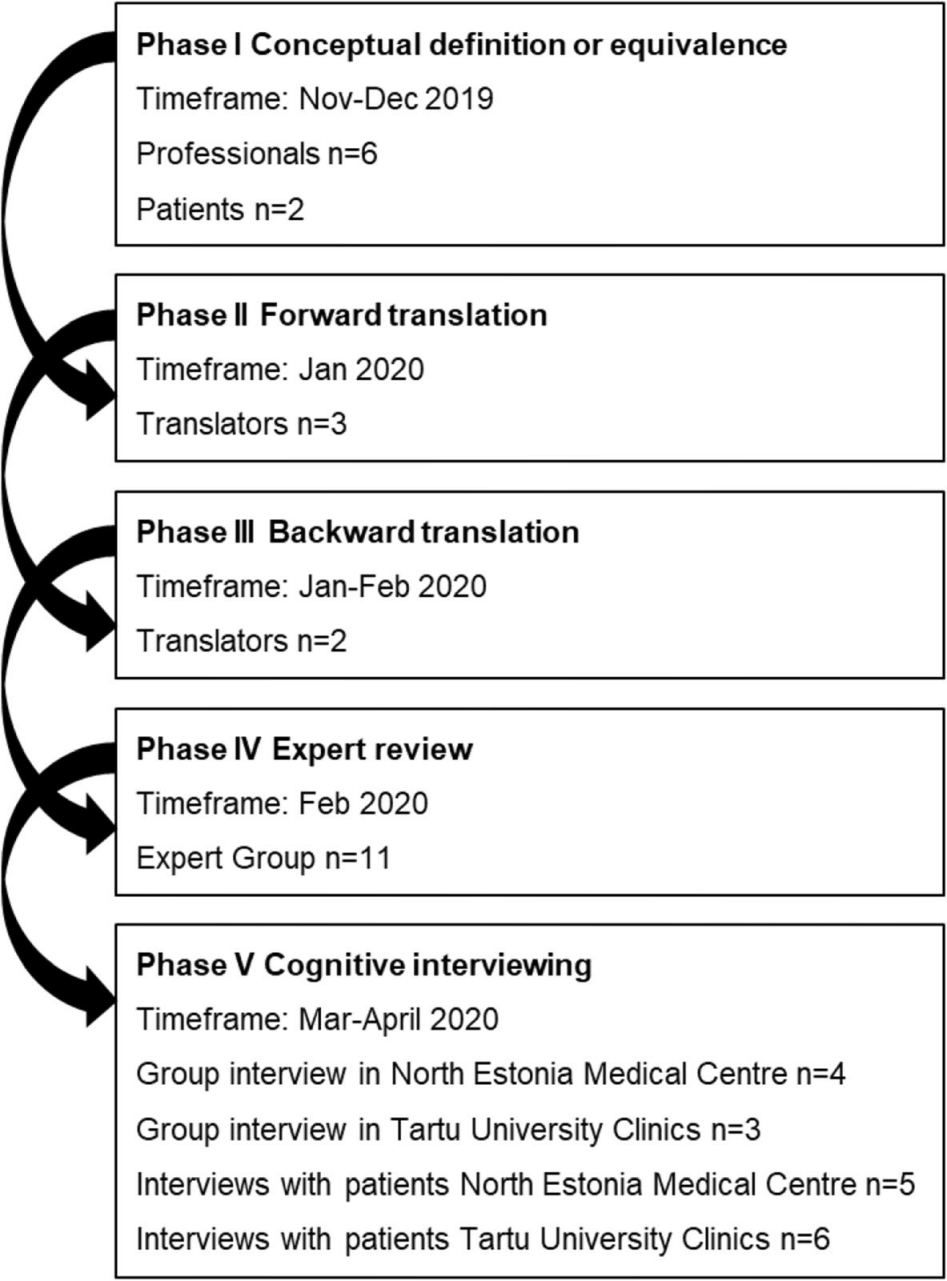
Overview of the study conduct and phases in the translation and cultural adaptation of IPOS in Estonia. Source: author, based on The POS author adaption manual [17]

POS author’s six phase guideline was used to translate and adapt the questionnaire [17]. The study took place from October 2019–May 2020 and was conducted in Tartu University Hospital (TUH) and North Estonia Medical Centre (NEMC) (Fig.1). The process included multiple methods such as forward translation-back translation method, expert focus group (*n* = 11) and cognitive interviews with PC patients (*n* = 11) from different PC settings and PC specialists (*n* = 8). Involved PC patients were recruited from different PC settings- home care, hospital’s day treatment centre and hospital. Analysis was done by using a thematic analyses [18] method.

The POS author adaption manual [17] is based on the generally recognised best practises for translation and cross-cultural adaptation and have been used in similar studies conducted for IPOS translation and cultural adaptation [6, 13–15, 19]. Psychometric testing was not performed within this study because according to the POS author’s manual [17] the recommended number of patients for psychometric testing is 150 to 200 which was not feasible as part of this study due to resource and time constraints. Studies conducted so far have indicated that the forward translation-back translation method in conjunction with cognitive interviews is sufficient for producing a content valid measure that can be successfully used in clinical practice [6, 13, 15, 20].

### Phase I conceptual definition and equivalence

As a first step, a literature overview to assess and evaluate the existing translations and adaptations of questionnaires and health-related quality of life issues in PC patients in Estonia was carried out. The literature overview was conducted by researcher ML using bibliographic search via PubMed database in October 2019 (with keywords used: *health-related quality of life in palliative care, palliative care, Estonia, PRO* and *PROM*) and screening of archive of local medical journal “Eesti Arst”. Retrieved articles (*n* = 4) were evaluated based on screening question: is the article about of PC or health-related quality of life in PC. Step two was a discussion on the relevance of IPOS measure items (conceptual definition, i.e. identification of key terms and concepts) with target population- PC professionals and PC patients in the form of an informal discussion. Discussions with professionals (*n* = 6) were conducted during the period of October–November 2019 and involved PC professionals from the Tartu University Hospital and the North Estonia Medical Centre. Discussions with PC patients (*n* = 2) were performed in November–December 2019. Discussions remained anonymous, and no patient data was collected.

### Phase II forward translation

Three translators were included in the forward translation process. Translator 1 (with a medical background) translated the measure to Estonian producing forward translation 1 (FT1). Translator 2 (with no medical background) translated the measure to Estonian producing forward translation 2 (FT2). Translator 3 synthesised forward translations FT1 and FT2 into forward translation 3 (FT3).

### Phase III Back translation

Two translators were involved in the back translation to English. Both translators were blinded to the original version of IPOS. Back translator 1 (with medical background) translated forward translation FT3 back to English, producing back translation BT1. Back translator 2 (with no medical background) translated forward translation FT3 back to English (without knowledge of or seeing the original English text), producing back translation BT2.

### Phase IV expert group review

An Expert Group meeting was performed via teleconference. Participants who could not attend the meeting sent their feedback before the meeting via email and their remarks and suggestions were considered during the Expert Group meeting. The Expert Group included translators participating in the translation process and PC professionals from both participating hospitals (Table 1). Researchers ML and RH were facilitators. Based on the synthesis of back-translated measures BT1 and BT2, one final back translation BT3 and a pre-final version of IPOS questionnaire in Estonian (patient and staff versions) was agreed on during the Expert Group detailed review and discussion panel.

**Table 1:** Expert Group members

Expert Group member	Profession	Role in the study
Clinician/expert group member	Physician	PC-Palliative care specialist
Clinician/expert group member	Physician	PC-Palliative care specialist
Clinician/expert group member	Physician	PC-Palliative care specialist
Cancer Centre Quality Manager	Nurse	PC-Palliative care specialist
Forward translator 1/expert group member	Physician/Translator	Translator
Backward translator 1/expert group member	Nurse/Translator	Translator
FT1&FT2 mediator/expert group member	Pharmacist	Translator
Forward translator 2/expert group member	English teacher	Translator
Backward translator 2/expert group member	Translator	Translator
Expert group participant/facilitator	Nurse	Student/Researcher
Expert group participant/facilitator	Health care professional	Supervisor/Researcher

Source: author

### Phase V cognitive interviews with PC patients and specialists

After the translation process described above, cognitive interviews with a convenience sample from the target population were conducted for pre-testing the measure. Face to face cognitive interviews (*n* = 2) and cognitive interviews via phone call (*n* = 9) were performed with PC patients (*n* = 11) followed by two face to face group interviews, with members of the multidisciplinary PC team in Tartu University Hospital (clinical psychologist, physician, nurse) and North Estonia Medical Centre (clinical psychologist, social worker, physician, chaplain). Phone interviews instead of face to face interviews were conducted due to restrictions due to COVID-19 outbreak in Estonia. Interviewees’ characteristics are reported in Table 2. During interviews, the cognitive interview method “Think out loud” [21] was used to assess how interviewees comprehend questionnaire instructions and items. This method has been successfully used for conducting cognitive interviews in prior questionnaire translation and adaptation studies [14, 15, 20].

**Table 2:** Patient demographics and clinical data

Type of patients	PC-Palliative care setting	Age	Gender
Oncology	Hospital	85	Female
Hematology	Home care	78	Female
Oncology	Hospital’s day treatment centre	45	Female
Oncology	Hospital’s day treatment centre	41	Male
Oncology	Hospital’s day treatment centre	50	Male
Oncology	Hospital	55	Male
Oncology	Hospital	69	Female
Oncology	Hospital	62	Female
Oncology	Hospital	60	Female
Oncology	Hospital	71	Female
Oncology	Hospital	67	Female

Source: author

#### Setting and participants

Study subjects were PC patients from the hospitals and home care setting along with the hospital’s multidisciplinary team members managing PC patients (physicians, nurses, social worker, chaplain, clinical psychologists) with a good comprehension of Estonian. Participant gathering was done by a PC physician via patient inclusion and exclusion criteria. Patients included in the study were at least 18 years old, speaking Estonian as the first language, receiving PC, provided verbal consent to participate in the study, capable of independently reading an informational leaflet on the study and the IPOS questionnaire, able to verbally explain how understands the items during a cognitive interview. Exclusion criteria for patients were as follows: unable to provide cognitive feedback on the questionnaire, or were terminally ill.

#### Study procedures

Prior to cognitive interviews, patients were given an informational leaflet explaining the reason for conducting the study and asked for verbal consent. Patients were asked to complete the IPOS pre-final version (either three days or seven days) and invited to “Think out loud” [21]**.** Patients were asked how they understood the instructions and items and verbalise their thoughts and understanding of the item as much as possible. If the patient believed the item was confusing or unambiguous, were asked to suggest a rewording. Interviews were recorded on an audio tape and later transcribed. PC teams were asked for verbal consent before group interviews. During interviews, the multidisciplinary PC team members were provided with staff versions of the questionnaire (three days and seven days) and asked to “Think aloud” [21] and verbalise their thoughts and understanding of the items as much as possible. Interviews were recorded on an audio tape and later transcribed.

### Phase VI- approval of the IPOS development team (proofreading)

The final IPOS patient and staff versions in Estonian with all the appendices and reports of entire process were submitted to the IPOS development team at Cicely Saunders Institute of Palliative Care, Policy & Rehabilitation Florence Nightingale Faculty of Nursing, Midwifery & Palliative Care King’s College London (POS team) for proofreading.

### Data analyses

Recorded interviews were transcribed using the Tallinn University of Technology speech transcription system for Estonian speech [22]. The results were collected for each question separately, coded and conclusions based on input from patients and group interviews were compared. Parts that were important for the study were re-transcribed and cleaned. Transcription analysis was done by Researcher ML.

## Results

### Findings and results during phase I conceptual definition and equivalence

The results from the performed literature overview showed that minimal information is available regarding PC and HRQL issues within PC patient population in Estonia (4 articles in the final inclusion sample, consisting of 2 articles from PubMed database and 2 articles from local medical journal “Eesti Arst”). The literature overview and initial specialist discussion concluded that no nationally accepted holistic measure or method is used or has been translated for the assessment of PC patient outcomes. In the initial discussion with the study target subjects- palliative care patients and specialists pointed out that the concepts defined in IPOS are well recognized and considered appropriate in Estonia.

### Findings and results during phase II- forward translation of the IPOS to Estonian

No major discrepancies between FT1 and FT2 were found. Challenges were noted on several occasions. For example, Q2 term *drowsiness* can be translated to Estonian as *dizziness (“uimasus”* in Estonian*)* or *sleepiness* (*“unisus”* in Estonian). Term *“uimasus”* was decided to be the most adequate. Translation of the scale items *occasionally* and *sometimes* were difficult to differentiate as both words sound univocal in Estonian and can be translated as *“harva”, “mõnikord”, “aeg-ajalt”* or *“vahetevahel”.* Occasionally was translated as *“harva”* and sometimes as *“vahetevahel”.* One of the forward translators questioned whether to translate throughout the questionnaire word “You” (in Estonian “Teie”) with a capital initial letter or not. Both options are acceptable in Estonian, though the initial capital letter is mostly used in formal correspondence. Considering the context and the aim of a questionnaire was decided to use the *“teie”* without using a capital letter.

### Findings and results during phase III- back translation of the IPOS to English

Both back-translations were reviewed and compared, and no problematic translations discovered, only one minor grammar discrepancy, which was then adjusted. Nevertheless, multiple items in the back-translations were not close to the original IPOS, and a few items were close, but not identical. These items were discussed in more details within the Expert Group meeting.

### Findings and results during phase IV- expert group review

During the Expert Group (*n* = 11) detailed review, the discussion was synthesised into one final back- translation BT3 based on the back-translated measures BT1 and BT2 and agreed on pre-final version of IPOS questionnaire in Estonian (patient and staff versions). Discussion in the expert group enabled to choose the most appropriate wording. Multiple modifications were implemented to the pre-final version of the measure and corresponding back-translation as a result of expert group review. Items Q4, Q7, Q8, Q10 were found to be correct (back-translation was identical with original IPOS [9] as well as translation to Estonian) and didn’t require additional modification. Findings highlighted that assessment of spiritual wellbeing “Q6. Have you felt at peace”- was considered unusual and an odd question by several patients and severely ill patients may not want to answer to this question. During expert group review, the original question in Estonian was revised to “Have you felt inner peace” which was tested during cognitive interviews. Further details of modifications implemented are given in Table 3. Final BT3 was sent to the IPOS development team at Cicely Saunders Institute of Palliative Care, Policy & Rehabilitation Florence Nightingale Faculty of Nursing, Midwifery & Palliative CareKing’s College London for the review and approval for continuation. Approval via email to continue with cognitive interviews was received.

**Table 3:** Issues and discrepancies identified and revised during an Expert Group review (*n* = 11)

Original English IPOS question/item	Discussion within Expert Group	Expert Group resolution
Period of time – *Over the past 3 days/week* within a questionnaire	Both *“during”* and *“over”* of the past 3 days/week were used in back translations.	BT3 – use *“over”* as also in original IPOS.
*Q2 scale item 4 “Overwhelmingly”*	Item was translated as “*äärmiselt tugevalt*”- it was discussed and proposed that *“väga tugevalt”* would be more appropriate to express the *“overwhelmingly* “in Estonian.	Translation of IPOS in Estonian and back translation modified. Translation to Estonian changed (from “*äärmiselt tugevalt*”- to “*väga tugevalt*”). Because of this change also the back translation modified (from “*extremely strongly”* to “*very strongly”*) to deliver the meaning the “*overwhelmingly”* in Estonian.
*Q2 listed symptom “Shortness of breath”*	Item was translated as *“hingeldus*”- it was discussed that *“õhupuudus”* would express more clearly the “*shortness of breath*”.	Translation of IPOS in Estonian modified (from “*hingeldus*” to “*õhupuudus*”) to deliver clearly the meaning the of “*shortness of breath”* in Estonian.
*Q2 listed symptom “Nausea (feeling like you are going to be sick)”*	Original IPOS has an additional note *(feeling like you are going to be sick)* added in brackets, which is not appropriate in Estonian context. *“Iiveldus”* is a clear definition and additional clarification in brackets is not needed it would be rather confusing.	Translation of IPOS in Estonian modified (definition in brackets removed as N/A), because of this also back translation modified accordingly (text in brackets removed).
*Q2 listed symptom “Poor appetite”*	Item was translated as *“isutus”* which can also mean no appetite at all. It was discussed that *“söögiisu vähenemine*” would be more correct translation to express the *“Poor appetite”.*	Translation of IPOS in Estonian modified (from *“isutus”* to *“söögiisu vähenemine*”). Because of this change also the back translation modified (from “*loss of appetite”* to “*decreased appetite”*).
*Q2 listed symptom “Poor mobility”*	Item was translated as *“raskendatud liikumine”* during the meeting was discussed that *“liikumisraskused”* would be more accurate term for *“Poor mobility”* in Estonian.	Translation of IPOS in Estonian changed from *“raskendatud liikumine”* to *“liikumisraskused”.* Because of this change also back translation changed from *“difficulty in moving”* to *“moving difficulties”.*
*Scale: 2 Sometimes*	Item was translated as *“vahetevahel”*. Same item was challenging also during FT. During the meeting was discussed that *“aeg-ajalt”* would express better the meaning of *“sometimes”* in Estonian.	Translation of IPOS in Estonian changed (from *“vahetevahel”* to *“aeg-ajalt”* to better deliver the meaning of the *“sometimes”* in Estonian.
*Q3. Have you been feeling anxious or worried about your illness or treatment?*	It was noticed and discussed that back translation did not include word *“feeling”.*	No changes needed- translation in Estonian is correct and question is well understood so no further modification required.
*Q5. Have you been feeling depressed?*	It was noticed and discussed that back translation did not include the word *“feeling”.*	No changes needed- translation in Estonian is correct and question is well understandable so no further modification required.
*Q6. Do you think s/he has felt at peace?*	The question was translated as *“Kas arvate, et ta on olnud rahulik?”* It was discussed that translation is too direct and rather express the physical status, not spiritual wellbeing and feeling of being at peace.	Several suitable terms were discussed. It was agreed that *“sisemine rahu”*- in English *“inner peace”* express the question most correctly in Estonian. Both patient and staff versions in Estonian and back translation were updated. The question in patient version re-worded from *“Kas arvate, et ta on olnud rahulik”* to *“Kas arvate, et ta on tundnud sisemist rahu?”* Back-translation modified from “*Do you think he/she has been calm?”* to *“Do you think s/he has felt inner peace?”*
*Q6. Have you felt at peace?*	Question was translated as *“Kas olete olnud rahulik?"* It was discussed that translation is too direct and rather express the physical status not spiritual wellbeing and feeling of being at peace.	Question modified from from *“Kas olete olnud rahulik?"* to *“Kas olete tundnud sisemist rahu?”* Back translation modified from *“Have you been calm?” to “Have you felt inner peace?”*
*Q9. Have any practical problems resulting from your illness been addressed? (such as financial or personal*) *3 Problems hardly addressed*	Item has been translated as *“probleemidega on vähesel määral tegeletud”-* it was discussed that *“probleemidega on vähe tegeletud”* would be clearer sentence.	Translation of IPOS in Estonian *“Probleemidega on vähesel määral tegeletud?*” changed to *“Probleemidega on vähe tegeletud”.*
*If you are worried about any of the issues raised on this questionnaire then please speak to your doctor or nurse*	During the discussion and review was pointed out that translation to Estonian *“Kui olete mures selles küsimustikus tõstatatud probleemide pärast, palun rääkige oma arsti või meditsiiniõega”* is missing *“any*”.	Translation of IPOS in Estonian modified from *“Kui olete mures selles küsimustikus tõstatatud probleemide pärast, palun rääkige oma arsti või meditsiiniõega”* to *“Kui olete mures ükskõik millise küsimustikus tõstatatud probleemi pärast, palun rääkige oma arsti või meditsiiniõega”.* Back translation modified from *“If you are worried about the issues raised in this questionnaire, please talk to your doctor or nurse”* to *“If you are worried about any of the issues raised in this questionnaire, please talk to your doctor or nurse”.*

Source: author, except the Original IPOS question/item (Palliative care Outcome Scale (POS). https://pos-pal.org/maix/)

### Findings and results during phase V- cognitive interviews with patients and PC specialists

The specialists interpreted the staff versions of questionnaire well (comprehension and judgment of the pre-final IPOS in Estonian: 9/9 questions assessed as very good or good), questions were considered easily understandable and appropriate. Detailed results of the staff comprehension and judgment of the pre-final IPOS are shown in Table 4.

**Table 4:** Results from cognitive group interviews with staff (*n* = 7)

ccx	Staff comprehension and judgment of the pre-final IPOS in Estonian
Q1. What have been the patient’s main problems or concerns over the past week?	Very good comprehension.
Q2. Please tick one box that best describes how the patient has been affected by each of the following symptoms over the past week? 0 Not at all 1 Slightly 2 Moderately 3 Severely 4 Over-whelmingly Cannot assess (e.g. unconscious) Pain Shortness of breath Weakness or lack of energy Nausea (feeling like you are going to be sick) Vomiting (being sick) Poor appetite Constipation Sore or dry mouth Drowsiness Poor mobility	In overall good comprehension. It was pointed out that the scale item- cannot assess (e.g. patient unconscious) is translated to Estonian “*ei saa hinnata (nt patsient ei ole teadvusel*)” which in Estonian may lead to an opinion that this box should be marked only when it is not possible to assess the status due to patient condition (e.g. patient unconscious). The question was raised if this box can still be marked if the specialist is not able to answer due to limited information (patient inadequate) or limited feedback from the patient. It was discussed and proposed to add additional word *(“ei oska”*) to Estonian term which would make it clearer “*ei saa/ei oska hinnata* (*nt patsient ei ole teadvusel)”.* Verb *“can”* is used in English in much broader sense compared to verb *“saama”* in Estonian. Some team members also proposed to add additional word *“inadequate”* to *“unconscious*”. Very good comprehension of listed symptoms.
Please list any other symptoms and tick one box to show how you feel each of these symptoms has affected the patient over the past week.	Very good comprehension.
Over the past week:	Very good comprehension.
Q3. Has s/he been feeling worried about his/her illness or treatment? 0 Not at all 1 Occasionally 2 Sometimes 3 Most of the time 4 Always Cannot assess (e.g. unconscious)	Very good comprehension. One participant asked if this question should be asked from the patient, as asking may cause additional anxiety and impression that something is wrong *(“mostly patients are worried, if no worry at all then patient might be inadequate”; “I would prefer sometimes not too ask if patient is anxious by the nature”).*
Q4. Have any of his/her family or friends been anxious or worried about the patient? 0 Not at all 1 Occasionally 2 Sometimes 3 Most of the time 4 Always Cannot assess (e.g. unconscious)	Very good comprehension.
Q5. Do you think s/he felt depressed? 0 Not at all 1 Occasionally 2 Sometimes 3 Most of the time 4 Always Cannot assess (e.g. unconscious)	Good comprehension. One of staff member has asked additional question who should assess it.
Q6. Do you think s/he has felt at peace? 0 Always 1 Most of the time 2 Sometimes 3 Occasionally 4 Not at all Cannot assess (e.g. unconscious)	Very good comprehension of the question (“*I feel that I’m able to answer if I have been in contact with patient”; “Question is clear, but sometimes it might be complicated to assess it in clinical setting”; It might be difficult to answer*).
Q7. Has the patient been able to share how s/he is feeling with his/her family or friends as much as s/he wanted? 0 Always 1 Most of the time 2 Sometimes 3 Occasionally 4 Not at all Cannot assess (e.g. unconscious)	Very good comprehension. *(“This is very good question”; “It is important to map how patient is communicating with family”; Patients sometimes do not want to share their feelings with friends and family”).*
Q8. Has the patient had as much information s/he wanted? 0 Always 1 Most of the time 2 Sometimes 3 Occasionally 4 Not at all Cannot assess (e.g. unconscious)	Good comprehension of the question. Additional discussion and questions raised what information exactly should be asked and mapped. (“*What information is meant”; “Sometimes patients are lacking information relevant for everyday wellbeing* e.g. *they do not know where cafeteria is located in the hospital“; To assess patient information needs over the time could here be a box for comments”; I may not remember what were the information needs”*).
Q9. Have any practical problems resulting from his/her illness been addressed? (such as financial or personal) 0 Problems addressed/ No problems 1 Problems mostly addressed 2 Problems partly addressed 3 Problems hardly addressed 4 Problems not addressed Cannot assess (e.g. unconscious)	Good comprehension of the question. Additional question by whom the practical questions should be addressed was raised. A few interviewees pointed out that Scale items 2 “*Problems partly addressed*” in Estonian “*Probleemidega on osaliselt tegeletud*” and 3 *“Problems hardly addressed*”, in Estonian *“Probleemidega on vähe tegeletud*” are very similar and it might be complicated to distinguish the difference between scale items 2 and 3. *(“There are too many options”; It would be clearer if there is just 3 or 4 options instead of 5″; “Difficult to assess, patients have different priorities“; “Again, comment box would be good to map the practical problems”; “It would be clearer if under personal is also mentioned day-to-day coping”*).

Source: author, except the Original IPOS question/item (Palliative care Outcome Scale (POS). https://pos-pal.org/maix/)

During both group interviews it was pointed out that the scale item- *cannot assess (*e.g. *patient unconscious)* is translated to Estonian *“ei saa hinnata (nt patsient ei ole teadvusel)”* which in Estonian may lead to an opinion that this box should be marked only when it is not possible to assess the status due to patient condition (e.g. patient unconscious). The question was raised if this box can still be marked if the specialist is not able to answer due to limited information or limited feedback from the patient. It was discussed and proposed to add an additional word *(“ei oska”*) to Estonian term which would make it clearer *“ei saa/ei oska hinnata (nt patsient ei ole teadvusel)”. V*erb *“can”* is used in English in much broader sense compared to verb “*saama*” in Estonian. One of the team members also proposed to add additional word *“inadequate”* to *“unconscious”.* The question about spiritual wellbeing (Q6) was well understood, but some participants felt that it might not be easy to assess in a clinical setting. Comprehension of the question about information needs (Q8) and the question about practical problems being addressed (Q9) was good (no comprehension issues reported during interviews), but some participants suggested that the question could have a comment box to specify the patient information needs and source of the problems.

The interviews with patients demonstrated that patients interpreted the patient versions of the questionnaire well (comprehension and judgment of the pre-final IPOS in Estonian: 8/10 questions assessed as very good or good) Questions were considered mostly easily understandable and appropriate. Detailed results of the patients comprehension and judgment of the pre-final IPOS are shown in Table 5.

**Table 5:** Results from cognitive interviews with patients (*n* = 11)

Original English IPOS question/item	Patients comprehension and judgment of the pre-final IPOS in Estonian
Q1. What have been your main problems or concerns over the past week?	Very good comprehension. The question was well received and understood. *(“Question is simple and logical, I understand it well”*). All patients responded with personal problems (“*my main concern has been how to avoid coronavirus”; “how to cope financially”*) as well as health-related problems *(“weakness”, “nausea”; “vomiting”*). Some patients responded that there were no problems at all.
Q2. Below is a list of symptoms, which you may or may not have experienced. For each symptom, please tick one box that best describes how it has affected you over the past week. 0 Not at all 1 Slightly 2 Moderately 3 Severely 4 Over-whelmingly Pain Shortness of breath Weakness or lack of energy Nausea (feeling like you are going to be sick) Vomiting (being sick) Poor appetite Constipation Sore or dry mouth Drowsiness Poor mobility	Good comprehension.1/11 patient suggested adding a word *average* (how it has affected you in *average* over the week) as she felt that is difficult to assess the pain as it constantly changes during the day/week *(“Pain comes and goes, no one is suffering pain while in the hospital …*”). Very good comprehension of listed symptoms.
Please list any other symptoms not mentioned above, and tick one box to show how they have affected you over the past week	Very good comprehension by all patients.
Over the past week:	Very good comprehension.
Q3. Have you been feeling anxious or worried about your illness or treatment? 0 Not at all 1 Occasionally 2 Sometimes 3 Most of the time 4 Always	Good comprehension. 1/11 patients felt that this question contains two questions (illness and treatment) and would like to differentiate the answers *(“I’m happy with my treatment, and I fully trust my doctor, but the disease is something I cannot control, and it makes me anxious”*). Very good comprehension of scale items.
Q4. Have any of your family or friends been anxious or worried about you? 0 Not at all 1 Occasionally 2 Sometimes 3 Most of the time 4 Always	Very good comprehension. All patients had a very good understanding of the question *(“I understand the question well”; “Accurate wording, I understand it”*). Very good comprehension of scale items.
Q5. Have you been feeling depressed? 0 Not at all 1 Occasionally 2 Sometimes 3 Most of the time 4 Always	Very good comprehension by all patients *(“I would not wish to say it somehow differently”; “Correct and relevant question”; “I understand it”*). Very good comprehension of scale items.
Q6. Have you felt at peace? 0 Always 1 Most of the time 2 Sometimes 3 Occasionally 4 Not at all	Good comprehension. The question was found inappropriate by one patient *(“What is this? I do not want to answer. There is no need for inner peace- logical thinking is needed”*). 3/11 patients admitted that question is a little bit unusual for them, but they understand what is meant by this question and are able to answer *(“I have found a balance between my disease and everyday life”; “I feel that I’m calm, goes as it goes, worrying does not help”; “This is a good feeling, “I feel it when I’m in nature”*). Very good comprehension of scale items.
Q7. Have you been able to share how you are feeling with your family or friends as much as you wanted? 0 Always 1 Most of the time 2 Sometimes 3 Occasionally 4 Not at all	Good comprehension by all patients. 1/11patient mentioned that question is coinciding with question 4 *(“I feel this question is similar to Q4. It is about friends and family. My friends and family have been worrying about me and I have talked to them”*). Very good comprehension of scale items.
Q8. Have you had as much information as you wanted? 0 Always 1 Most of the time 2 Sometimes 3 Occasionally 4 Not at all	4/11 patient asked what kind of information is meant by the question (e.g. disease, treatment, nutrition etc.) and considered question too general *(“What kind of information is meant here”; “Information from what source”; “If here is meant information about the disease and /or treatment then clarification could be in brackets”; “Question is too general and wide-ranging, could be specified”*). Very good comprehension of scale items.
Q9. Have any practical problems resulting from your illness been addressed? (such as financial or personal) 0 Problems addressed/ No problems 1 Problems mostly addressed 2 Problems partly addressed 3 Problems hardly addressed 4 Problems not addressed	2/11 patients felt that question is not easy to comprehend; they had to read it twice and asked additional questions before answering *(“Could you please clarify”; “What is meant here”*), but did not suggest to re-sentence it. 2/11 patients pointed out that time window (week/3 days) is too short for assessment whether practical problems are addressed or not *(“Should I answer thinking about the last week?”; “There have been some problems and they were addressed, but earlier”*).
Q10. How did you complete this questionnaire? On my own With help from a friend or relative With help from a member of staff	Very good comprehension by all patients. Very good comprehension of scale items.
If you are worried about any of the issues raised on this questionnaire then please speak to your doctor or nurse	Very good comprehension by all patients.

Source: author, except the Original IPOS question/item (Palliative care Outcome Scale (POS). https://pos-pal.org/maix/)

Similar to staff group interview feedback, some comprehension difficulties were identified by patients in Q8 and Q9. The question about the information needs (Q8) was considered too general as patients asked clarification about the information source meant in the question. Q9 was considered difficult to understand as some patients had to read it twice and also asked additional information before answering. One of additional questions was about the time window (week/3 days) as for Q9 it was considered too short for some patients. In one case it was difficult for patient to assess the symptom pain over time (Q2) due to fluctuating nature of pain and adding a word *“average”* was considered. Another concern was related to the question about feeling anxious or worried about your illness or treatment (Q3) where patient felt that differentiation of the answers for treatment and disease could be done. The question about spiritual wellbeing (Q6) was not considered acceptable in one case where the patient refused to answer. Some patients admitted that this question is bizarre and not usual. However, they conceded that the concept is relevant and understandable and answered the question. The question about family or friends being anxious or worried about the patient (Q4) and able to share feelings with family and friends (Q7) were felt to be similar by one patient.

Overall acceptability of the pre-final versions of IPOS was good. Questionnaire was considered relevant and not burdensome to answer. Spontaneous comments included *(“I think it is a good questionnaire”; “useful”; practical questionnaire- no one has asked if I have received enough information and I do lack it”).*

### Findings and results during phase VI- proofread

Appendices and reports describing the whole translation and cultural adaptation process were submitted to IPOS team for proofreading. The final IPOS patient and staff versions in Estonian were approved by IPOS development team at King’s College London.

## Discussion

During this study, the IPOS patient and staff versions were translated and culturally adapted into Estonian. Face and content validity of the questionnaire was demonstrated by conducting cognitive interviews with patients and group interviews with PC specialists. The pre-final versions of IPOS patient and personnel questionnaires were deemed of good quality (comprehension and judgment of the pre-final IPOS in Estonian assessed as very good or good) by the cognitive interview process. Questionnaire was considered relevant and not burdensome to answer. No interviews were interrupted due to tiredness or difficulties.

Our experience of IPOS translation shows that similarly to a French [14] study, we identified several possible weaknesses in original questionnaire. For instance, Q2 assessment of fluctuating symptoms, Q3 binary options for illness or treatment, Q8 about source of information. However, the questions remained in accordance with the concepts in the original questionnaire.

Rigorous translation process enabled to produce the questionnaire that appears to be equivalent to the original measure. The two forward translations were largely consistent with one another. But several differences were found such as item *“drowsiness”* and Likert scale items “*occasionally”* and “*sometimes”.* With the help of third translator (mediator) consensus was achieved*.* Similarly to forward translations both backward translations were close to the original measure. However, in several occasions back translation did not match the original IPOS showing that direct translation from English to Estonian became misleading. These dissimilarities were discussed and resolved with Expert group*.* Following the translation, thorough expert group review identified several weak points and translation alternatives. The expert group identified that for certain concepts (e.g. spiritual wellbeing) direct translation from English to Estonian became misleading (it is not “being calm”) and required cultural adaption. Our study accented how difficult it can be to ask about spiritual wellbeing as this is considered very delicate and personal information that severely ill patients may not want to discuss about and staff may feel difficulties in assessing it.

The fact that our Expert Group consisted of translators with and without medical background and PC physicians and specialists working daily with PC patients proved to be very useful as due to diversity of experts background different angles of the questions including semantic variations were highlighted, discussed and finally consensually agreed.

Most of the interviews (9/11) with patients were conducted over the phone and no differences were identified compared to face-to-face interviews. Results show that IPOS explores domains in different settings and age groups. No differences were identified between different settings (collecting points of the data), mode or neither from age groups.

However, we have encountered similar problems in patient interviews, as reported by other researchers. Difficulties in assessment of fluctuating symptoms was raised during the previous research done in Italian [15] and UK/German [20] study. Similarly to Italian [15] study, we found that patients may get confused when a question is double-barreled e.g. Q3. which is about anxiety or worry about the illness and treatment.

Similarly to study conducted in Sweden [13], where was pointed out that Q8 about information needs was confusing to Swedish patients was Q8 confusing to Estonian patients. A few patients expressed a surprise what information should be provided and considered question too general. Q9 about practical problems being addressed was challenging both to patients and staff. Some patients had to read it twice before answering, but did not suggest to re-sentence it, just admitted that question is somehow complicated. Some interviewed staff members felt that under personal could be also mentioned day-to-day coping and it would ease the assessment. Also, question raised an overall discussion about the needed awareness level from staff perspective e.g. how much staff should know about the patient’s personal problems.

Analyzing the translation and cultural adaptation process was realized that importance of following the precise methodology cannot be under estimated to produce a validated and culturally adapted translation of the questionnaire [6, 10, 13–15, 20].

This study has several limitations including a relatively small sample size. However, the sample size was in accordance with IPOS development team guidance [17] and similar work conducted [12–15, 20].

Most patients interviewed had cancer, which limit the generalizability of study results to patients suffering from other life-threatening illnesses. One researcher conducted both group and patient interviews and was responsible for the analyzes of the transcriptions, possibly leading to some bias. To ensure that this bias did not happen the research team lead supervised the work done by the main researcher.

The majority of patient interviews were conducted over the phone due to COVID-19 outbreak restrictions (hospitals were in quarantine and visits not allowed).

## Conclusions

The IPOS Patient and Staff versions were translated and culturally adapted for Estonia. Recommended guidelines were used to create Estonian versions equivalent to the original measures. The Estonian IPOS in its four versions, demonstrated face and content validity and are ready for clinical use in Estonia.

## Data Availability

The interview transcripts generated and analyzed during the current study are available on reasonable demand to corresponding Author.

## Abbreviations

APCA POS: African Palliative Care Association Palliative Care Outcome Scale
BT: Back translation
FT: Forward translation
IPOS: Integrated Palliative care Outcome Scale
POS: Palliative care Outcome Scale
PC: Palliative Care
PROM: Patient Reported Outcome Measure
PRO: Patient Reported Outcome
HRQL: Health Related Quality of Life
